# Software compatibility analysis for quantitative measures of [^18^F]flutemetamol amyloid PET burden in mild cognitive impairment

**DOI:** 10.1186/s13550-023-00994-3

**Published:** 2023-05-24

**Authors:** Hugh G. Pemberton, Christopher Buckley, Mark Battle, Ariane Bollack, Vrajesh Patel, Petya Tomova, David Cooke, Will Balhorn, Katherine Hegedorn, Johan Lilja, Christine Brand, Gill Farrar

**Affiliations:** 1 GE Healthcare, Pollards Wood, Chalfont St Giles, Amersham, HP8 4SP UK; 2 Centre for Medical Image Computing (CMIC), Department of Medical Physics and Bioengineering, University College London, London, UK; 3 UCL Queen Square Institute of Neurology, University College London, London, UK; 4 Clinical Memory Research Unit, Department of Clinical Sciences, Lund University, Malmö, Sweden; 5 Syntermed, Atlanta, GA USA; 6 MIM Software, Cleveland, OH USA; 7 HERMES Medical Solutions, Stockholm, Sweden

**Keywords:** Amyloid PET, SUVr, Alzheimer’s, MCI, Quantification, [^18^F]flutemetamol

## Abstract

**Rationale:**

Amyloid-β (Aβ) pathology is one of the earliest detectable brain changes in Alzheimer’s disease pathogenesis. In clinical practice, trained readers will visually categorise positron emission tomography (PET) scans as either Aβ positive or negative. However, adjunct quantitative analysis is becoming more widely available, where regulatory approved software can currently generate metrics such as standardised uptake value ratios (SUVr) and individual *Z*-scores. Therefore, it is of direct value to the imaging community to assess the compatibility of commercially available software packages. In this collaborative project, the compatibility of amyloid PET quantification was investigated across four regulatory approved software packages. In doing so, the intention is to increase visibility and understanding of clinically relevant quantitative methods.

**Methods:**

Composite SUVr using the pons as the reference region was generated from [^18^F]flutemetamol (GE Healthcare) PET in a retrospective cohort of 80 amnestic mild cognitive impairment (aMCI) patients (40 each male/female; mean age = 73 years, SD = 8.52). Based on previous autopsy validation work, an Aβ positivity threshold of ≥ 0.6 SUVr_pons_ was applied. Quantitative results from MIM Software’s *MIMneuro*, Syntermed’s *NeuroQ,* Hermes Medical Solutions’ *BRASS* and GE Healthcare’s *CortexID* were analysed using intraclass correlation coefficient (ICC), percentage agreement around the Aβ positivity threshold and kappa scores.

**Results:**

Using an Aβ positivity threshold of ≥ 0.6 SUVr_pons_, 95% agreement was achieved across the four software packages. Two patients were narrowly classed as Aβ negative by one software package but positive by the others, and two patients vice versa. All kappa scores around the same Aβ positivity threshold, both combined (Fleiss’) and individual software pairings (Cohen’s), were ≥ 0.9 signifying “almost perfect” inter-rater reliability. Excellent reliability was found between composite SUVr measurements for all four software packages, with an average measure ICC of 0.97 and 95% confidence interval of 0.957–0.979. Correlation coefficient analysis between the two software packages reporting composite z-scores was strong (*r*^2^ = 0.98).

**Conclusion:**

Using an optimised cortical mask, regulatory approved software packages provided highly correlated and reliable quantification of [^18^F]flutemetamol amyloid PET with a ≥ 0.6 SUVr_pons_ positivity threshold. In particular, this work could be of interest to physicians performing routine clinical imaging rather than researchers performing more bespoke image analysis. Similar analysis is encouraged using other reference regions as well as the Centiloid scale, when it has been implemented by more software packages.

## Introduction

The distribution of brain amyloid-beta (Aβ) can be measured using positron emission tomography (PET). Three Fluorine-18 radiolabelled Aβ PET tracers have been approved for clinical use: [^18^F]flutemetamol (Vizamyl™; GE Healthcare) [1], [^18^F]florbetaben (Neuraceq™; Life Molecular Imaging) [2] and [^18^F]florbetapir (Amyvid™) [3]. Clinical appraisal of amyloid PET imaging involves binary classification (Aβ negative or positive) through visual assessment, which has been demonstrated as approximately 90% accurate in advanced clinical and end-of-life patients [1, 2, 4]. Over the last two decades, multiple studies have demonstrated the clinical utility of amyloid PET [5–15]. In addition, real-world studies have shown that an amyloid PET scan can increase diagnostic confidence [5, 9, 12, 14, 15], change etiological diagnosis in 25–44% of cases [5, 8, 9, 16] and change patient management in 37–72% of cases [8–10, 12].

However, in recent years memory clinics are increasingly assessing ‘pre-dementia’ patients, with ~ 25% of cases presenting with subjective cognitive decline (SCD) or early mild cognitive impairment (MCI) [17]. In these patients, amyloid deposition might be focal or early-stage [18], which may confound visual assessment, especially by less experienced readers [19]. In these cases, the binary classification approach may be more prone to subjectivity given the reliance on the clinician’s prior experience, possibly resulting in greater inter-rater variability [3, 20–23]. Adjunct quantitative measures of Aβ deposition, such as Standardized Uptake Value ratio (SUVr) [24], may also bring clinical benefit for early assessment [11, 25–27]. SUVr quantifies the ratio of tracer uptake between a reference region and a target region, when the radiotracer is estimated to have reached pseudo-equilibrium [24]. Furthermore, quantification could provide greater clinical utility alongside current dichotomous classification, such as improvements in diagnostic confidence [8, 10, 21], prediction of cognitive decline [28–31] and changes to diagnosis [16] and patient management [32–37].

Amyloid PET quantification has been used in research since the discovery of Carbon-11 labelled Pittsburgh compound B ([^11^C]PiB), in 2004 [24, 38]. This has resulted in several sophisticated examples of research software for processing and quantifying amyloid PET, such as PMOD, CapAIBL [39] NiftyPET [40], EvaLuation of Brain Amyloidosis (ELBA) [41], AmyPype [42] and rPOP [43]. Concurrently, various regulatory approved (FDA 510k/CE-marked Class IIa) software packages have been designed for use in clinic, yet none are currently in widespread use for amyloid PET quantification. Clinical use of amyloid PET in the USA relies upon visual inspection, however it is interesting to note that the 2020 SNMMI Value Initiative “National Amyloid Survey” found 52% of sites (out of 176 surveyed with amyloid imaging experience) were using adjunct quantification software. The translational paucity among the other half of respondent may be down to a number of factors, but a lack of clinical validation is likely to contribute [44]. One aspect of the current work is to demonstrate the compatibility of software tools to ensure generalisability amongst users.

Therefore, this validation study aimed to investigate composite SUVr from a group of clinically relevant patients with amnestic MCI (aMCI) across four regulatory approved software packages, and measure the concordance of these quantitative results. In doing so, the secondary aim is to increase visibility and understanding of clinically available quantitative methods. The use of the composite SUVr measure was recently endorsed by the recent RSNA QIBA profile as a relevant and logistically feasible measure for amyloid quantification [45]. Each software package has unique individual features, but this collaborative project brought competing vendors together to demonstrate the concordance of results using a single relevant measure when a composite mask was implemented, and to facilitate use of quantification in routine clinical assessment. The hypothesis was that after the harmonisation exercise, all software packages under investigation will provide highly correlated quantitative results (composite SUVr) according to kappa scores, and pairwise/groupwise correlation.

## Methods

### Patient and scan information

All patients included in this analysis had previously participated in Institutional Review Board (IRB), Independent Ethics Committee (IEC)-approved studies and they (or their legally authorized representative) provided informed written consent to participate; data usage and image analysis was considered to be covered by the previous consent.

This retrospective study included two analysis phases: a pilot and a validation phase. Images for each phase were taken from patients who had participated in previous development studies for [^18^F]flutemetamol. The pilot data comprised of imaging sets from 11 patients from the phase II ALZ201 study [46]. The larger validation data (*n* = 80 validation data, 40 each male/female; mean age = 73 years) comprised of images from aMCI patients who had taken part in a Phase III clinical trial which determined the proportions of normal and abnormal images and the prediction of future clinical progression relative to amyloid status [47]. In both studies, patients received a single dose of approximately 185 MBq (range 166–203 MBq) of [^18^F]flutemetamol with image acquisition starting ~ 90 min (range 85–95 min) after injection, and collected 6 × 5 min frames. Baseline clinical features of the 80 aMCI subjects included measurement of MMSE (mean = 27), CDR (0.5) and, Activities of Daily Living (mean = 74) [47].

### Software packages

Four regulatory approved software packages (see Table 1) were used to generate composite SUVr from [^18^F]flutemetamol:

- MIM Software’s *MIMneuro* (https://www.mimsoftware.com/nuclear_medicine/mim_neuro)
- Syntermed’s *NeuroQ* (https://www.syntermed.com/neuroq)
- Hermes Medical Solutions’ *BRASS* (https://www.hermesmedical.com/neurology/)
- GE Healthcare’s *CortexID* (https://www.gehealthcare.com/courses/aw-cortex-id)

**Table 1:** Summary of four regulatory approved software packages and associated features

–	MIM software’s *MIMneuro*	Syntermed’s *NeuroQ*	Hermes medical solutions’ *BRASS*	GE healthcare’s *CortexID*
CE/FDA status	CE Mark and FDA 510(k)	CE Mark and FDA 510(k)	FDA 510(k)	FDA 510(k)
Imaging requirements	PET, optional MRI or CT	PET, optional MR or CT	PET, optional MRI or CT	PET, optional MRI or CT
Normative database	54 controls	25 controls	80 controls [48]	> 100 controls
QC procedure	Visual QC for registration quality			
Partial volume correction	Not performed			
Spatial normalisation	Standard space (MNI)			
Attenuation correction	Required for image acquisition and processing			
Reference regions	Customizable e.g. pons, whole cerebellum, cerebellar cortex	Pons, whole cerebellum	Pons, whole cerebellum, cerebellar cortex	Pons, whole cerebellum, cerebellar cortex
Target regions available in addition to harmonised mask	Customizable e.g. Left/right hemisphere for: anterior cingulate gyrus, inferior medial frontal gyrus, lateral temporal lobe, posterior cingulate gyrus, precuneus, and superior parietal lobule	Left/right hemisphere for all (except midbrain and vermis): frontal cortex, sensorimotor cortex, broca’s region, anterior cingulate, posterior cingulate, caudate nucleus, lentiform nucleus, thalamus, parietal cortex, parietotemporal cortex, lateral temporal cortex, medial temporal cortex, primary visual cortex, associative visual cortex, midbrain, and vermis	Left/right hemisphere for all: frontal cortex, anterior cingulate, occipital cortex parietal cortex, lateral temporal cortex, precuneus/posterior cingulate,	Left/right hemisphere for all: Prefrontal, anterior cingulate, precuneus/posterior cingulate, parietal, lateral/mesial temporal, occipital, sensorimotor
Metrics reported	SUVr, z-score, Centiloid*	SUVr, z-score	SUVr, z-score	SUVr, z-score

*May not be approved in all jurisdictions

### Normative database demographics

- *Cortex ID* The [^18^F]flutemetamol normal database of 100 amyloid negative scans spans the age range from 30 to 85 years with the majority of the subjects being 55 years old and above. Amyloid load as measured by amyloid PET in amyloid negative controls has a very weak association with subject age and thus no age correction of the normal database was deemed necessary
- *BRASS* database of 80 subjects is a subset of the 100 contained in *CortexID*
- *NeuroQ* normal data base consists of [.^18^F]flutemetamol scans from 25 cognitively unimpaired subjects (10 < 55 years and 15 > 55 years) [46]
- *MIMneuro* The [^18^F]flutemetamol normal database contains 54 exams from AIBL. Exams were classified as normal according to AIBL criteria and have a negative amyloid scan upon visual assessment. Ages span from 60 to 84 years old.

### Image processing

All images were processed within the graphical user interfaces (GUI) of:

- *BRASS* using *v2.10.1.0,* by HP/PT (GE Healthcare, Amersham, UK). The *BRASS* GUI loads in DICOM folders, the correct tracer/reference region must be selected and then registration initiated. Registrations were visually checked and quantitative results were then exported for analysis.
- *CortexID* using *v2.1 Ext. 6,* by VP (GE Healthcare, Marlborough, USA). The CortexID GUI requires DICOM import, image registration automatically follows and quality was checked visually. SUVr results were then exported for analysis.
- *MIMneuro* using *vMIM-7.2.1 LA21-01,* by WB/KH (MIMSoftware, Ohio, USA). The MIMneuro GUI requires DICOM import. The tracer is detected automatically from DICOM headers, and the reference region is selected based on the tracer. Registration is both rigid and deformable. Registrations were checked visually and results exported for analysis.
- *NeuroQ* using *v3.80,* by HP/PT (GE Healthcare, Amersham, UK). The NeuroQ GUI requires import of patient’s DICOM, and selection of reference region and appropriate tracer database. Both rigid and nonlinear registration were then performed and visually quality checked. Next, composite SUVr was generated and databased.

**Fig. 1:**
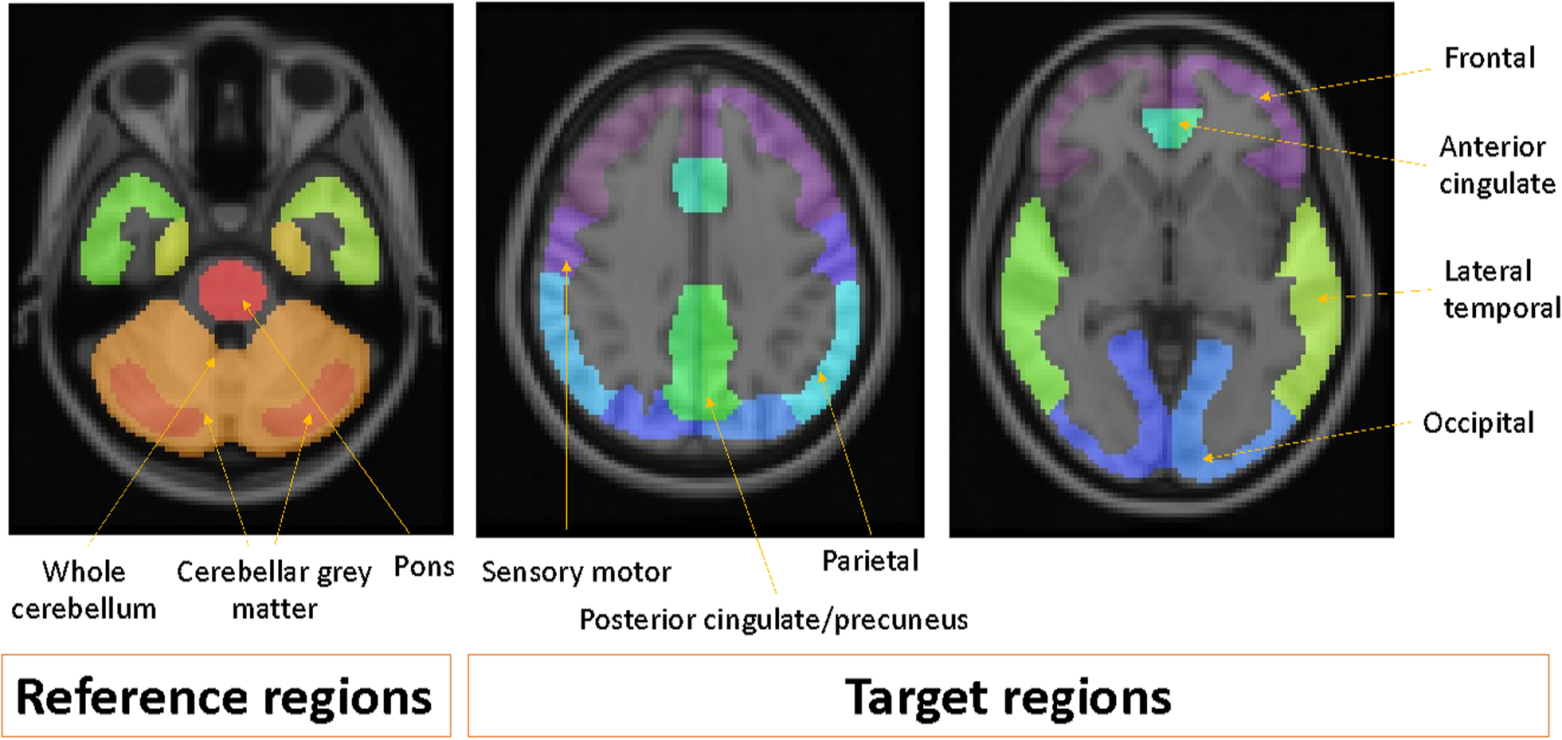
Representation of the cortical mask optimised for the measurement of [^18^F]Flutemetamol

For the purposes of both the pilot and validation phases, the pons was used as the reference region for all measures as it is an autopsy validated reference region where the positivity threshold has high concordance with a large cohort of visually inspected [^18^F]flutemetamol images [46, 49]. Composite SUVr was generated from cortical volumes of interest of the following regions: prefrontal, anterior cingulate, precuneus/posterior cingulate, parietal, lateral/mesial temporal, occipital, sensorimotor, see Fig. 1. This figure displays the cortical mask developed by Thurfjell et al. [49] for the purpose of an optimised quantitation of [^18^F]flutemetamol. All reference regions are displayed but, as previously mentioned, only the pons was used as the reference region for analyses in the present study paper; although the SUVr results for both whole cerebellum and cerebellar grey are minimally less (by 1–4%) when compared against autopsy verified images and those with visual inspection results [49].

### Pilot data processing

Using a pilot dataset of 11 patients with varying amyloid load obtained from the aforementioned Phase II ALZ201 study [46], an estimate of concordance (percentage agreement around ≥ 0.6 SUVr positivity threshold) was obtained using a composite SUVr generated from the pre-existing cortical masks of each of the four software packages. In this pilot phase, varying agreement was observed across the software packages, likely due to heterogeneous cortical masks. Therefore, the cortical mask used to generate the composite cortical SUVr for *CortexID* was shared with all other software packages, with the aim of harmonising composite measures of amyloid burden. Once shared, the cortical mask was implemented into the pipelines of all software packages*,* and the pilot data was reassessed. Design of the mask is based on previous autopsy validated work [49] where care was taken to minimise any interference from white matter signal, which may potentially compromise any resulting SUVr measures. The CortexID cortical mask used for this harmonisation exercise is now available for other pipelines to use and can be obtained by contacting either GE Healthcare or the corresponding author.

### Validation data processing

Upon completion of pilot testing it was agreed with all vendors to further assess a larger validation data set. Composite SUVr was generated with all four software packages from reconstructed and attenuation corrected images from 80 aMCI patients of varying amyloid load [47]. Images were checked visually for quality of registration and segmentation. For the purpose of this project, the pons was used as the reference region for all measures as this has previously been shown a stable reference region [46]. Additionally, SUVr measures using the pons have been added to the [^18^F]flutemetamol summary of product characteristics in the EU (https://www.ema.europa.eu/en/documents/product-information/vizamyl-epar-product-information_en.pdf). Therefore, use of the pons as a reference region allows the development of datasets which are consistent with recommended routine clinical use.

### Statistical analysis

All 80 aMCI images were processed and quantified using four software packages. The composite SUVr values were analysed to assess compatibility. Percentage agreement was calculated across all software packages using an Aβ positivity threshold of ≥ 0.6 SUVr, derived from previous autopsy confirmation work [49]. In order to assess reliability among the software packages, group-wise correlation (intraclass correlation coefficient, ICC) on composite SUVr was measured for all software packages combined. Kappa scores were calculated to assess inter-rater reliability of binary clinical decision between each pair of software packages (Cohen’s) and group-wise (Fleiss’). Company names have been blinded when reporting results in order to avoid any bias as this was a standardisation exercise and not competitive positioning. Finally, Bland–Altmann plots were generated to visually compare the agreement per patient between each pair of software packages. Statistics were performed using R for Mac version 1.2.5036.

### Assessment of SUVr(pons) values relative to visual inspection

Majority visual read data (from 5 blinded readers) from a previous study [46] was available for the 80 aMCI cases examined in this study. The agreement rates between the majority and individual readers relative to the SUVr (pons) results from the 4 software tools, once the cortical masks had been implemented, is reported since this method of interpretation is also recommended in the European Summary of Product Characteristics (SmPC).

## Results

As this study has been a collaborative and non-competitive effort to promote standardisation of [^18^F]flutemetamol analysis software names have been anonymised when reporting results.

### Pilot data compatibility

**Fig. 2:**
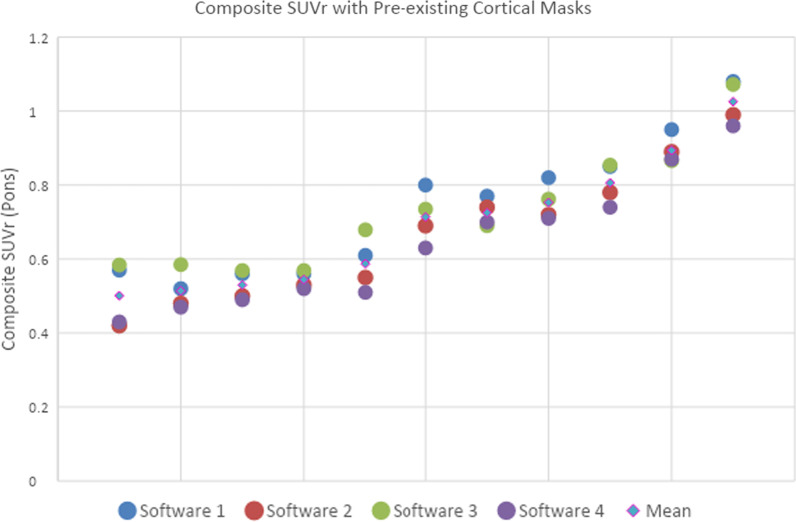
Composite SUVr of 11 patients (4 dots per patient) from pilot data using the pons as reference region with pre-existing cortical masks for all four software packages, the data is ranked by the patient’s mean SUVr across the four software packages

**Fig. 3:**
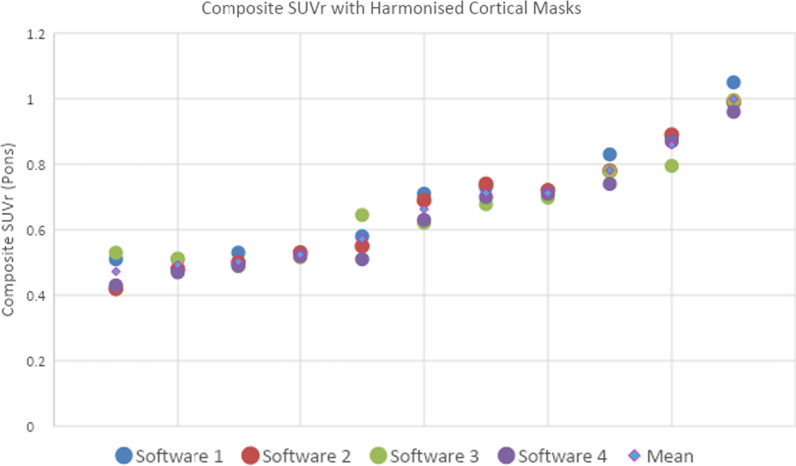
Composite SUVr of 11 patients from pilot data using the pons as reference region with harmonised cortical mask for all four software packages, the data is ranked by the patient’s mean SUVr across the four software packages

Initial analysis of the composite SUVr for the 11 patients in the pilot phase showed good agreement between two of the software packages and marginally less with the other two, see Fig. 2 for graph and Table 2 further below for numeric results. The cortical masks from Softwares 1 and 3 were more heterogeneous, which lead to some differences in the agreement between the composite SUVr, see Table 2 below. Subsequently, the cortical mask from *CortexID* was shared with all vendors, incorporated into the respective analysis pipelines and results reprocessed (see Fig. 3 and Table 2). Analysis of all four software packages found a 95% agreement around a ≥ 0.6 SUVr positivity threshold.

**Table 2:** Mean composite SUVr (*n* = 11 pilot patients) of four regulatory approved software packages with the original cortical masks, harmonised cortical masks, followed by the mean difference (∆) between Software 3/Software 1 and Software 2 with original and harmonised cortical masks

–	SW 2 Original	SW 4 Original	SW 4 ∆ SW 2 Original	SW 3 Original	SW 3 ∆ SW 2 Original	SW 3 Harmonised	SW 3 ∆ SW 2 Harmonised	SW 1 Original	SW 1 ∆ SW 2 Original	SW 1 Harmonised	SW 1 ∆ SW 2 Harmonised
Mean composite SUVr	0.663	0.639	− 0.024	0.724	0.061	0.660	− 0.003	0.735	0.073	0.689	0.026

*SW* software. Software names are anonymised due to proprietary nature of the software and potential commercial implications

### Pilot data with harmonized cortical mask

Figure 3 shows the agreement across the 4 software packages for the composite SUVr once the cortical masks had been harmonised across processing pipelines. An overall percentage agreement of 98% was calculated around an Aβ positivity threshold ≥ 0.6 SUVr.

Table 2 shows the composite SUVr for all software packages using both the original and the harmonised cortical masks. In this pilot phase, good initial agreement was observed between two software packages’ composite SUVr (mean SUVr difference of -0.024). However, harmonising the cortical mask across all four vendors improved consistency by reducing the difference in mean composite SUVr for:

- Software 3 vs Software 2
  - original masks = 0.061
  - harmonized masks = -0.003, a 0.064 reduction in mean composite SUVr difference
- Software 1 vs Software 2
  - original masks = 0.073
  - harmonized masks = 0.036, a 0.047 reduction in mean composite SUVr difference

Following this improvement step, it was agreed with all vendors to further assess a larger (*n* = 80) validation data set (see “Validation data”).

Note, the overall the net variation of the harmonised quantitation values (Table 2) are at a similar level to that observed in test re-test studies, which was 0.9–3.8% [46].

### Validation data

#### Reliability

**Fig. 4:**
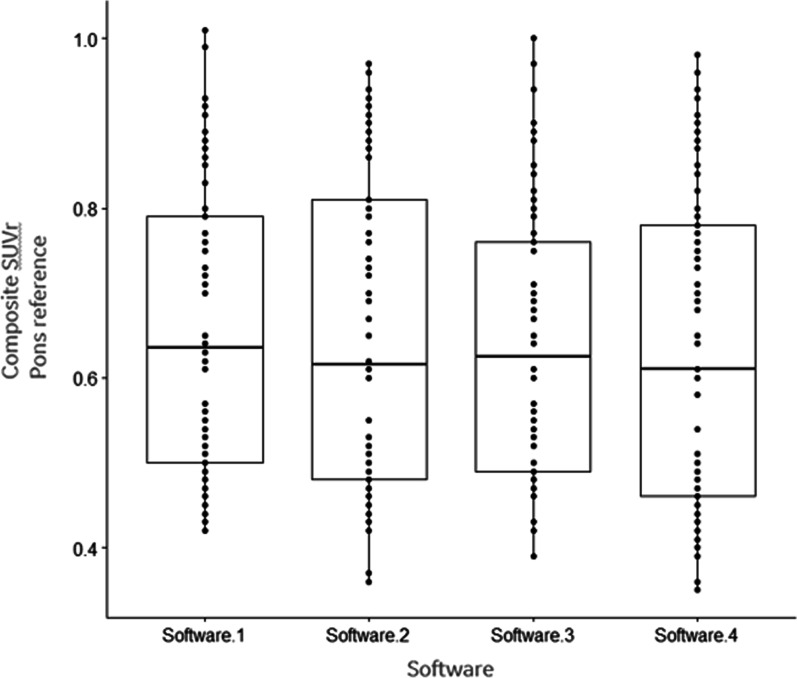
Boxplot showing composite SUVr using the pons as a reference region for each of the 4 software packages analysed

The average measure ICC was 0.97, 95% confidence interval from 0.957 to 0.979, denoting ‘excellent’ reliability between composite SUVr measurements for all four software packages, see Fig. 4 showing boxplots for each software.

#### Kappa scores around binary threshold classification

All kappa scores around the ≥ 0.6 SUVr Aβ positivity threshold, both combined (Fleiss’) and individual pairings (Cohen’s), were ≥ 0.9 signifying “almost perfect” inter-rater reliability. Fleiss’ Kappa score for the four software packages together = 0.95. See Table 3 for the pair-wise Cohen’s Kappa score for each of the software package combinations.

**Table 3:** Cohen’s Kappa score for all possible software pairs

Software package pair	Cohen’s Kappa
1 vs 2	1
1 vs 3	0.92
1 vs 4	0.97
2 vs 3	0.92
2 vs 4	0.97
3 vs 4	0.90

Software names are anonymised due to proprietary nature of the software and potential commercial implications

#### Agreement

Using an Aβ positivity threshold of ≥ 0.6 SUVr [49], 95% agreement was achieved across the software packages. Two patients were narrowly classed as *negative* by one software package but *positive* by the others, and two patients vice versa, see Table 4 for composite SUVr of the 4 discordant patients.

**Table 4:** Summary of four discordant patients around a ≥ 0.6 SUVr positivity threshold, bold values indicate results disagreeing on amyloid status with the other three software packages

Discordant patient	Composite SUVr			
	Software 1	Software 2	Software 3	Software 4
#1	0.61	0.61	**0.57**	0.61
#2	0.51	0.48	**0.65**	0.46
#3	0.62	0.62	0.61	**0.58**
#4	0.52	0.51	**0.64**	0.51

#### Assessment of SUVr(pons) values relative to visual inspection

Majority visual read data (from 5 blinded readers) from a previous study [46] was available for the 80 aMCI cases examined in the present study. Visual read Kappa score was 0.89 (95% CI 0.82–0.96). By majority read, 43 cases were positive and 37 were negative. 72/80 cases were concordant by all 5 readers; some discordancy was observed in 8 cases (7 negatives with 2 calling positive in 3 cases and 1 calling positive in 4 cases, and a single positive case where one reader called a negative). In the present study, SUVr(pons) at a threshold of 0.6 differentiated the visual read cases in a dichotomous pattern, where only 3/80 for software 3 and × 1/80 cases for software 4 were discordant, and thus where the visual inspection and software result might have led to a discordant analysis. In cases such as these it is recommended that in clinical use there is a subsequent reassessment of the image more closely to see if there are any potential artefacts (e.g. image atrophy, lesions in the reference region or ROI) that may support whether the reader finalises their result based upon either the visual or quantitative analysis.

#### Z-score analysis

**Fig. 5:**
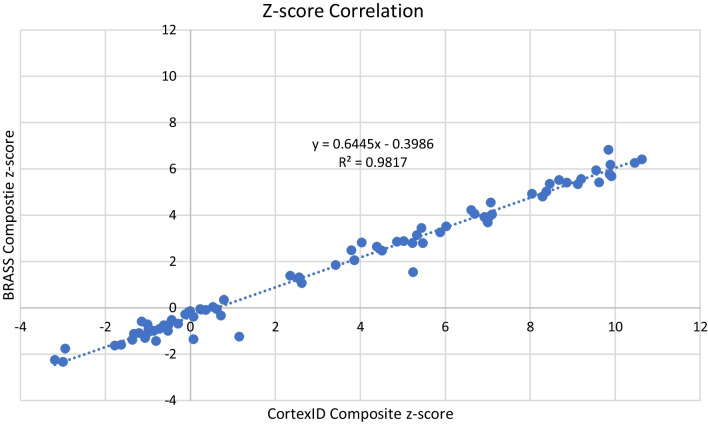
*Z*-score correlation analysis between the only two software packages assessed in this paper which offer composite z-scores

Of the four software packages assessed in this paper, only Cortex ID and Hermes report a composite Z-score. The resulting correlation coefficient analysis demonstrates a strong (*r*^2^ = 0.98) linear relationship between the composite z-score analysis of the 80 aMCI subjects for the two software packages, see Fig. 5.

### Bland–Altmann plots

**Fig. 6:**
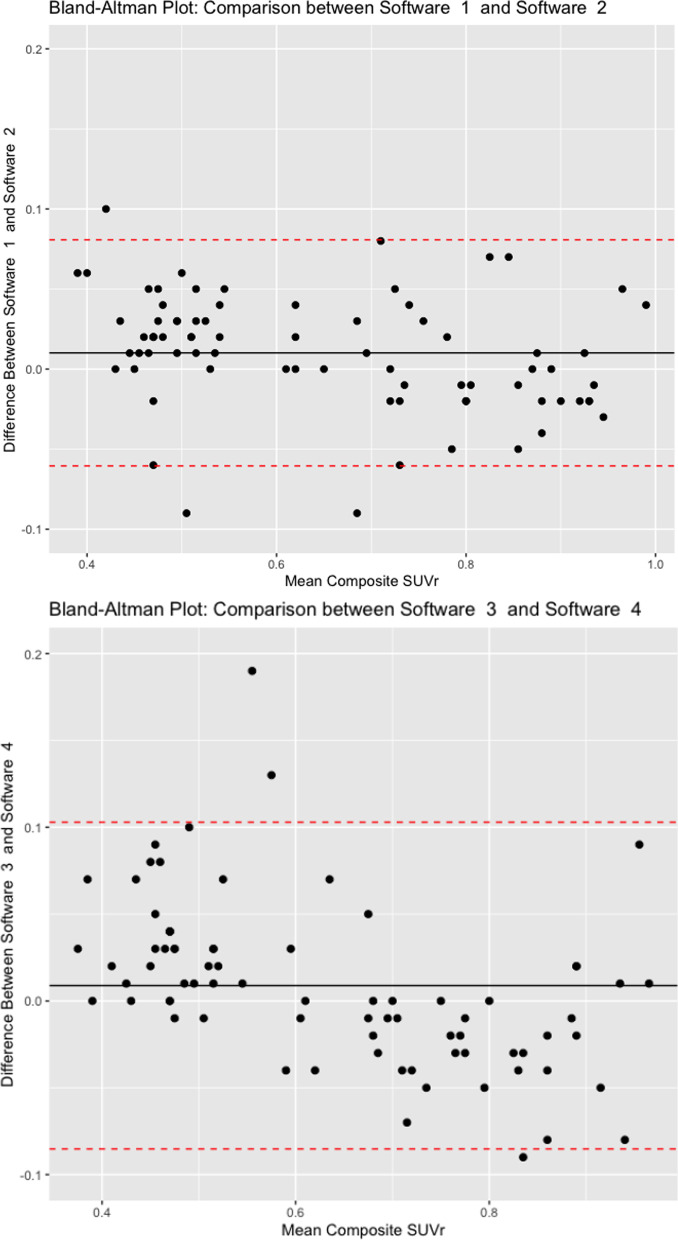
Bland–Altmann plots for the highest (Software 1 and 2) and lowest (Software 3 and 4) agreement scores, according to Cohen’s kappa. Y-axis shows the difference between the composite SUVr for each software package and the x-axis shows the mean composite SUVr of the two software packages

Bland–Altmann plots display the relationship between two paired variables using the same scale, i.e. composite SUVr. The black dots show the average measurement of the 2 software packages in question, the black line shows the average difference in measurements between the two software packages and the red dotted lines show the upper and lower limits of the 95% confidence interval (CI) for the average difference between the two software packages. Figure 6 shows Bland–Altmann plots for the highest (Software 1 and 2) and lowest (Software 3 and 4) agreement scores, according to Cohen’s kappa. The top plot shows tighter 95% CI and smaller average difference in measurements between the two software packages.

## Discussion

In this work, four regulatory approved software packages have generated highly correlated and reliable quantification (composite SUVr) of [^18^F]flutemetamol amyloid PET around a ≥ 0.6 SUVr Aβ positivity threshold [49], using the pons as a reference region. All kappa scores around this threshold, both combined (Fleiss’) and individual pairings (Cohen’s), were ≥ 0.9 signifying “almost perfect” inter-rater reliability; the average measure ICC was 0.97. Strong concordance was achieved through harmonisation of cortical masks for generating quantitative amyloid PET results. This was possible due to the collaborative efforts among competing software vendors, all of whom have now implemented to the cortical mask examined in the present study.

The clinical standard of binary classification through visual assessment has been demonstrated as approximately 90% accurate in advanced clinical and end-of-life patients [1, 2, 4]. This provides useful stratification of amyloid status for research, clinical trials and routine clinical practice. However, visual assessment can be challenged in a heterogeneous clinical population. For example, cortical thinning or atrophy can be compounded by partial volume effects, which subsequently raises the question of performing partial volume correction (PVC), or not. There is no consensus in the field regarding this issue; some recent evidence suggests an increase in sensitivity for detecting early stage cerebral amyloidosis when using PVC [50]. However, other studies comparing various methods have proven inconclusive [51, 52]. It is worth noting none of the software packages in the study currently perform PVC. Comorbidities further complicating visual assessments include normal pressure hydrocephalus [53] or other neurodegenerative disorders [13, 37, 54–56]. Adjunct quantitative measures of Aβ deposition, such as those examined in this study, can provide greater clinical utility in addition to current dichotomous classification and contribute to improvements in diagnostic confidence [8, 10, 21], prediction of cognitive decline [28–31] and changes to diagnosis [16] and patient management [32–37].

Previous work has been carried out comparing regulatory approved software packages (Hermes and Syngo.via) on 225 subjects (probable AD, MCI, controls), showing high sensitivity and specificity for both [47]. Most recently, Syngo.via, CortexID and PMOD were assessed on 195 patients with cognitive impairment, marginally different positivity thresholds were noted along with very high correlation between different software and normalization methods [57]. The present study has compared twice as many software packages approved for clinical use (i.e. FDA 510(k) cleared and/or CE-marked and demonstrated very strong concordance across all four applications. The focus for the larger validation set was on clinically relevant subjects (i.e. aMCI patients), in line with the amyloid PET appropriate use criteria [58], i.e. patients with “persistent or progressive unexplained MCI”, and perhaps more likely to benefit from amyloid PET in the earlier stages of cognitive impairment. It is worth noting that visual inspection, even after quantification, is still recommended in order to assess cases which may have atrophy and where quantification may potentially be compromised.

As noted in Table 1, the software packages have a variety of features and image processing differences. While the results were highly compatible across all four software packages following harmonisation of the cortical mask, there was disagreement in 5% of cases (*n* = 4) where two patients were narrowly classed as positive by one software package but negative by the others, and two patients vice versa. Possible reasons for these minor discrepancies are differences in spatial normalization and registration steps carried out by each vendor. In addition, despite harmonisation of the cortical regions, the pons reference region may not be equally harmonized, and thus could contribute to the minor discrepancies observed. However, as a high-uptake region, the pons is likely to be more robust to such variations than other reference regions. However, the reliability results from this validation exercise (Table 2) demonstrate that the variation is approximately comparable to that observed in test–retest [46] and that measurement of [^18^F]flutemetamol using a harmonised cortical mask would not influence analysis above that observed for test-rest.

Only Cortex ID and Hermes report a composite Z-score, the correlation coefficient analysis was strong (see Fig. 5). It is worth noting that the threshold between negative and positive scans for the two packages differs: Cortex ID has a threshold of approximately 2 whilst that of Hermes is lower at approximately 1.5. These differences are likely due to the composition and size of the normative databases, as shown in Table 1.

### Limitations

Ideally, all four software packages would have been installed on the same workstation and results independently generated. However, due to the proprietary nature of the software this was not possible and three of the software packages were installed on different workstations at GE Healthcare with the final vendor generating their own results before sharing for group-wise analysis. In addition, only composite SUVr with the pons as reference region was assessed; use of other reference regions and quantitative metrics was beyond the scope of planned work.

Pons was used as the reference region in this analysis since the aim was to generate data to support the language in the European Summary of Product Characteristics (SmPC), which has recently been updated to add quantitation as an adjunct to visual inspection of [^18^F]flutemetamol images. Stated thresholds using pons as the reference region in the SMPC are quoted to be 0.59–0.61 and are derived from autopsy validated images using CERAD pathology as the standard of truth. Other reference regions can be used and in addition to pons but data showing the concordance between quantitation and visual inspection was 1–2% less than that derived from the pons [49].

It is also appreciated that other quantitative metrics, such as z-score, may be useful alternatives/adjuncts to the SUVr measure. A cortical z-score of over 2 is normally used to indicate whether the composite uptake of PET amyloid is abnormal [49]. Possibly more relevant for the z-score measure is to use this metric to assess early regional amyloid uptake when the composite measure is close to, or at, the threshold [59].

### Future directions

The Centiloid scale is a cross-tracer SUVr transformation which produces a single-figure for amyloid burden measure, which is expected to be consistent across tracers. While the method currently provides global rather than regional amyloid measures, clinical use of the Centiloid scale is increasing [59]. Therefore, as more software packages begin to offer this metric as part of the suite, similar compatibility analysis is encouraged. Further analysis of the more subtle differences between the processing pipelines of the four software packages in question was beyond the scope of this paper. However, additional analysis would be of interest to further elucidate the root cause of the minor discrepancies observed. It would also be of note to assess the compatibility of regional SUVr, in addition to the composite measures in the current paper. The z-score analysis in the paper encourages further investigation into the potential value of a consolidated normative database assessing the impact of database size and composition on the z-score threshold value.

## Conclusions

Regulatory approved and/or cleared software packages provide highly correlated and reliable quantification of [^18^F]flutemetamol amyloid PET based around a ≥ 0.6 SUVr positivity threshold, when using pons as a reference region. This concordance was achieved through collaboration between competing vendors, and the harmonisation of cortical masks for generating quantitative amyloid PET results. Where possible, harmonisation of image processing steps is encouraged in order to facilitate clinical validation and widen adoption of clinically relevant quantitative measures, with the ultimate aim of enhancing consistency of image interpretation leading to accurate diagnosis and management decisions in patients with AD pathology.

## Data Availability

The datasets used and/or analysed during the current study are available from the corresponding author on reasonable request.
